# Efficient Band Structure Calculation of Two-Dimensional Materials from Semilocal Density Functionals

**DOI:** 10.1021/acs.jpcc.1c02031

**Published:** 2021-05-13

**Authors:** Abhilash Patra, Subrata Jana, Prasanjit Samal, Fabien Tran, Leila Kalantari, Jan Doumont, Peter Blaha

**Affiliations:** † School of Physical Sciences, National Institute of Science Education and Research, HBNI, Bhubaneswar 752050, India; ‡ Institute of Materials Chemistry, Vienna University of Technology, Getreidemarkt 9/165-TC, Vienna A-1060, Austria

## Abstract

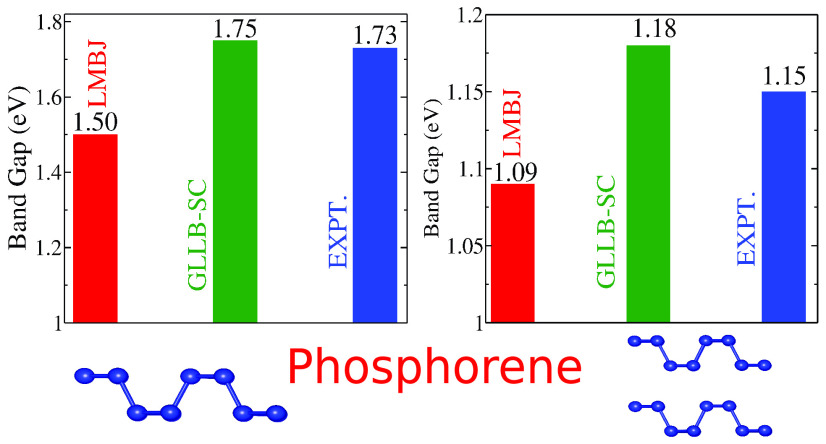

The experimental and theoretical realization of two-dimensional (2D) materials is of utmost importance in semiconducting applications. Computational modeling of these systems with satisfactory accuracy and computational efficiency is only feasible with semilocal density functional theory methods. In the search for the most useful method in predicting the band gap of 2D materials, we assess the accuracy of recently developed semilocal exchange–correlation (XC) energy functionals and potentials. Though the explicit forms of exchange–correlation (XC) potentials are very effective against XC energy functionals for the band gap of bulk solids, their performance needs to be investigated for 2D materials. In particular, the LMBJ [J. Chem. Theory Comput.2020, 16, 26543209700410.1021/acs.jctc.9b01147] and GLLB-SC [Phys. Rev. B82, 2010, 115106] potentials are considered for their dominance in bulk band gap calculation. The performance of recently developed meta generalized gradient approximations, like TASK [Phys. Rev. Res.1, 2019, 033082] and MGGAC [Phys. Rev. B. 100, 2019, 155140], is also assessed. We find that the LMBJ potential constructed for 2D materials is not as successful as its parent functional, i.e., MBJ [Phys. Rev. Lett.102, 2009, 2264011965888210.1103/PhysRevLett.102.226401] in bulk solids. Due to a contribution from the derivative discontinuity, the band gaps obtained with GLLB-SC are in a certain number of cases, albeit not systematically, larger than those obtained with other methods, which leads to better agreement with the quasi-particle band gap obtained from the *GW* method. The band gaps obtained with TASK and MGGAC can also be quite accurate.

## Introduction

The one-atom-thick layers of exfoliating materials have attracted the attention of the scientific community for their exceptional properties with many applications.^1,2^ The weak interaction between the layered bulk materials allows us to obtain the monolayer, and the most successful example is graphene,^3,4^ a single layer of graphite. The sp^2^-hybridized carbon atoms arranged in the hexagonal lattice in graphene lead to a very stable structure, and a free electron present at each site causes high charge mobility.^5^ Due to the semimetallic linear dispersion relation at the Fermi energy, graphene is not a good candidate for semiconductor devices. However, the band gap of graphene can be tuned by doping foreign atoms or saturating the extra electron with the termination of appropriate atoms.^6^ Besides graphene-based structures, other nanomaterials such as layers of transition-metal dichalcogenides (TMDs)^7^ or phosphorene (layered black phosphorus)^8−10^ have gained high interest for their intrinsic semiconducting band gap.^11^

The Kohn–Sham (KS) density functional theory (DFT),^12,13^ which is highly successful for total energy calculations,^14^ can also lead to accurate band gaps provided that an appropriate method for the exchange–correlation (XC) effects is used. For computational efficiency, one has to rely on the semilocal level of approximations, i.e., the local density approximation (LDA), the generalized gradient approximation (GGA), or the meta-GGA. Unlike the total-energy-related ground-state properties, the LDA, and most GGAs are inefficient in predicting the band gap, and their inefficiency is intrinsic to the KS eigenvalues they provide. In practice, the difference between the eigenvalues at the valence band maximum (VBM) and the conduction band minimum (CBM) is interpreted as the fundamental band gap. However, the fundamental band gap *E*_g_^KS^ obtained from explicit density-dependent methods (i.e., LDA/GGA), which lead to a multiplicative potential, deviates from the experimental value *E*_g_, defined as the ionization potential *I* minus the electron affinity *A*(*E*_g_ = *I* – *A*), by a quantity known as the XC derivative discontinuity (Δ_xc_)^15^
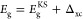
1In refs (16, 17) (see ref (18) for a recent summary), the problems of GGA-type XC energy functionals with respect to the derivative discontinuity and their performance in predicting band gaps are discussed. While Δ_xc_ = 0 with GGAs (therefore most of them clearly underestimate the band gap), meta-GGA XC energy functionals include Δ_xc_ to some extent when they are implemented in the generalized KS framework,^19^ i.e., by taking the derivative of the XC energy functional with respect to the orbitals instead of the density as in the KS method. The improvement of meta-GGAs over GGAs in calculating band gaps has been shown recently (see, e.g., refs (20−23)). XC semilocal methods that consist of only a potential (i.e., no energy functional can be associated) have been proposed in refs (24−28) and can be very successful in calculating the band gap of solids.^29−32^ Besides these semilocal methods, hybrid approaches that use a fraction of the exact Hartree–Fock exchange in combination with a semilocal XC functional are comparatively efficient for band gaps;^33−35^ however, the downside of the hybrid methods is that the accuracy in the band gap comes with a much higher computational cost. Similarly, calculating quasi-particle band gaps using the *GW* method^36^ is computationally extremely demanding. Furthermore, an overestimation by fully self-consistent *GW* in sp-bonded systems has been reported.^37,38^ Another method, DFT + *U*,^39^ also leads to more accurate band gaps, but can only be applied to localized electrons like in antiferromagnetic transition-metal oxides. Thus, to keep a balance between accuracy and computational cost, we should rely on the semilocal methods.

For the band gap of bulk solids, the semilocal potentials that are modeled directly can, in principle, be made much more accurate than the LDA or GGA potentials that are functional derivatives of energy functionals. Indeed, modeling directly the potential allows for much more flexibility in the choice of the analytical form. But their behavior on two-dimensional (2D) materials needs to be investigated more systematically.^40−42^ In ref (28), it was shown that the Gritsenko et al. (GLLB)^27^ exchange-only potential combined with the PBEsol correlation potential^43^ (GLLB-SC), modeled for solids, has a mean absolute error (MAE) smaller than the MAE obtained with the Heyd–Scuseria–Ernzerhof (HSE)^44,45^ hybrid functional for a set of 76 bulk solids.^30^ However, the mean absolute relative error (MARE) is smaller in the case of HSE. On the other hand, for a set of 472 solids, the MAE and MARE of GLLB-SC (0.7 eV and 39%) error are larger than the MAE of HSE (0.5 eV and 31%).^18^ By considering both the MAE and MARE, the modified Becke–Johnson (MBJ)^25^ potential is overall the best in predicting the band gaps for both sets of solids.^18^ Though MBJ is efficient for bulk calculations, the unit cell-dependent parameter present in the potential cannot be used in the case of systems with vacuum, like 2D materials or molecules. To address this problem, Rauch et al.^46^ proposed a model to bypass the unit cell-dependent function with a locally averaged function (see the Theory section for details), and the resulting potential is known as the local MBJ (LMBJ) potential. The application of LMBJ on a set of 298 2D materials has shown that the band gaps are of similar accuracy to HSE.^47^

However, the accuracy of the GLLB-SC and LMBJ potentials needs to be investigated further for 2D systems, like for instance, graphene-based systems, monolayer of TMDs, phosphorene layers, or MXenes, that are considered in the present work. Moreover, it will be interesting to compare and analyze these potentials against semilocal energy functionals for the above-mentioned systems. For this purpose, we have selected functionals that are well known or expected to lead to reasonable band gaps of solids^22,32^ and 2D materials.^6,48^ We will consider the Perdew–Burke–Ernzerhof (PBE)^49^ and high local exchange 2016 (HLE16)^50^ GGA-type energy functionals, the aforementioned LMBJ and GLLB-SC potentials, and four MGGA xc energy functionals: strongly constrained and appropriately normed (SCAN),^51^ high local exchange 2017 (HLE17),^52^ Aschebrock–Kümmel (TASK),^53^ and a functional recently proposed by Patra et al. (MGGAC).^54^ Along with these semilocal methods, we collected the band gaps obtained at different levels of the *GW* method and HSE hybrid functional. With the latter method, we calculated the band gap in the cases where no value is available in the literature.

The manuscript is organized as follows. It starts with the Theory section, which gives a description of the XC methods used in this work: potentials that are not functional derivatives of any energy functional (LMBJ and GLLB-SC) and meta-GGA functionals. Then, the computational details are given in the next section, followed by the discussion of the results. Finally, the Summary and Conclusions section summarizes our work and mentions possible future research.

## Theory

Details about the XC LMBJ and GLLB-SC potentials and the considered meta-GGA functionals are given below.

### MBJ and LMBJ Potentials

The MBJ potential is a modified version of the Becke–Johnson (BJ) potential that was originally proposed to reproduce the exact exchange optimized effective potential (OEP) in atoms.^24^ The modification was adopted to get improved band gaps for solids, which were only moderately improved with the original BJ potential.^55^ The form of the MBJ potential is given by
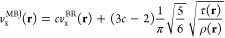
2where ρ = ∑_*i* = 1_^*N*^|ψ_*i*_|^2^ and τ = ∑_*i* = 1_^*N*^|∇ψ_*i*_|^2^/2 are, respectively, the electron density and kinetic energy density constructed from the occupied KS orbitals ψ_*i*_. The potential *v*_x_^BR^ is the Becke–Roussel (BR) exchange potential^56^ derived by generalizing the hydrogen exchange hole to other systems and given by
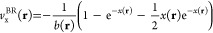
3where *b* = [*x*^3^ e^–*x*^/(4πρ)]^1/3^. The dimensionless quantity *x* is obtained by solving a nonlinear equation involving ρ, ∇ρ, ∇^2^ρ, and τ. The specificity of the MBJ potential given in eq 2 lies in defining the additional parameter *c* (for *c* = 1, the MBJ potential recovers the BJ potential). The second term present on the right-hand side of eq 2 is added to the BR potential to account for the difference between the OEP and the BR potential.^24^ The form of *c* was parameterized as
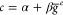
4with α = −0.012, β = 1.023 bohr^1/2^, *e* = 1/2, and
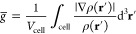
5is the average of |∇ρ|/ρ in the whole unit cell of volume *V*_cell_. The parameters α and β were determined by minimizing the MAE of the band gap for a set of 23 solids.

The problem with the MBJ potential arises in systems with vacuum (2D materials, surface structures, etc.), since the average given by eq 5 has no meaning. To address this issue, the local MBJ (LMBJ) potential was proposed in ref (46). The idea is to calculate *g̅* by averaging |∇ρ|/ρ locally instead of taking the average in the whole unit cell
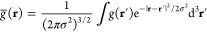
6where σ = 3.78 bohr is the smearing parameter. Rauch et al. decided to use the parameters α = 0.488, β = 0.5 bohr, and *e* = 1 in eq 4, which were determined in ref (57). The form of the function *g*, which was chosen by enforcing the constraint *c* → 1 in the low-density regime, is given by^46^
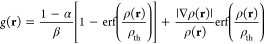
7The introduced threshold density was optimized by calculating the band gaps of a set of 22 semiconductors and set to ρ_th_ = 6.96 × 10^–4^*e*/bohr^3^, which corresponds to a Wigner–Seitz radius *r*_s_^th^ = ((4/3) πρ_th_)^−1/3^ = 7 bohr (see the erratum of ref (47)).

The LMBJ potential was successfully applied to interfaces and a large set of nonmagnetic 2D materials by Rauch et al.^46,47^ The error of the LMBJ potential is close to that of the HSE hybrid method, but with much less computational effort, which makes the LMBJ very interesting for assessing its accuracy further by applying to our selected systems.

### GLLB and GLLB-SC Potentials

The GLLB^27^ exchange potential is an approximated KS exchange potential that was modeled to get the shell structure of atoms similar to the OEP.^58^ In the GLLB method, the KS potential is separated into two parts
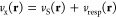
8where *v*_S_ is the Slater potential^59^ responsible for recovering the correct asymptotic behavior(*v*_S_(**r**) → −1/|**r**| for **r** → ∞). The additional potential *v*_resp_ arises from the integral of the linear response of the pair-correlation function. This second part of the potential is short-ranged repulsive, and the shell structure of atoms is mainly due to this part, as discussed in ref (27). The exact form of both these potentials in terms of pair-correlation function can be found in ref (27). The evaluation of *v*_S_ is computationally expensive; therefore, it is approximated by employing a GGA-type exchange energy functional. Since using an exchange energy functional with the correct asymptotic behavior −1/*r* is an advantage, Gritsenko et al.^27^ used the B88^60^ exchange functional to model *v*_S_, i.e., *v*_S_ in eq 8 is replaced by 2ε_x_^B88^, where ε_x_^B88^ is the exchange energy density per particle. In the GLLB potential, the second part of eq 8 is modeled with an approximation based on the idea from the Krieger–Li–Iafrate (KLI)^61^ simplification to the OEP. It was constructed by imposing three important constraints, namely, gauge invariance, scaling property of the potential, and exact recovery of the homogeneous electron gas (HEG) limit. It reads
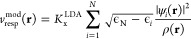
9where *K*_x_^LDA^ = 0.382 is fixed from the HEG and ϵ*_N_* is the highest occupied orbital energy of an *N* electron system. For applying the GLLB potential in solid-state systems, Kuisma et al.^28^ proposed to slightly modify GLLB by employing PBEsol^43^ instead of B88 for the exchange energy density ε_x_. Their second modification consists of adding the PBEsol correlation potential *v*_c_^PBEsol^ = δ*E*_c_^PBEsol^/δρ. Thus, the resulting potential, named GLLB-SC, reads
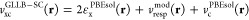
10The response potential *v*_resp_^mod^ (eq 9) is responsible for an abrupt change in the potential upon an infinitesimal occupation of the lowest unoccupied orbital. This leads to a derivative discontinuity given by^62^
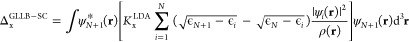
11where ψ_*N*+1_ is the lowest unoccupied orbital and ϵ_*N*+1_ is the corresponding eigenvalue.

The band gap calculations from Kuisma et al. with the GLLB-SC potential showed impressive results^28^ compared to standard GGA functionals, and the recent assessment of GLLB-SC on a large test set containing 472 bulk solids showed the strength of this potential in band gap calculation.^18^ In a recent study, it has been shown that GLLB-SC shows very good agreement with *G*_0_*W*_0_ for a subset of 2D materials of the C2DB database.^42^

### Advanced Meta-GGA Energy Functionals

The accuracy of meta-GGA functionals for various properties of solids is now well established.^51^ For instance, meta-GGA functionals are more accurate in predicting band gaps than GGAs^20^ thanks to the use of an additional ingredient, τ, and therefore to a nonzero derivative discontinuity when implemented in a gKS framework.^21^ A general form of the meta-GGA exchange energy functionals is written as
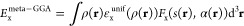
12where ε_x_^unif^(ρ) = −(3/(4π))(3π^2^ρ)^1/3^ is the exchange energy density per particle of the HEG, and *F*_x_(*s*, α) is known as the enhancement factor. It is a functional of the reduced density gradient *s* = |∇ρ|/[2(3π^2^)^1/3^ρ^4/3^] and the Pauli kinetic energy enhancement factor α = (τ – τ^W^)/τ^unif^, where τ^W^ = |∇ρ |^2^/(8ρ) is the von Weizsäcker kinetic energy density and τ^unif^ = (3/10) (3π^2^)^2/3^ρ^5/3^ is the kinetic energy density of the uniform electron gas.

As mentioned before, we consider four meta-GGA energy functionals, namely, SCAN,^51^ HLE17,^52^ TASK,^53^ and MGGAC,^54^ for their ability to be more accurate than the standard PBE in band gap calculations. To get a clear idea about the form of their enhancement factors, readers are recommended to follow the references mentioned above. The strength of the SCAN functional is that it satisfies all 17 known constraints imposed to a meta-GGA XC energy functional. On the contrary, HLE17^52^ was (empirically) constructed specifically for the band gap of solids and excitation energy of molecules by enhancing the TPSS^63^ exchange energy by 5/4 and reducing TPSS correlation energy by 1/2. The TASK exchange functional (combined with LDA correlation^64^) was constructed by redesigning the SCAN exchange with a stronger negative slope ∂*F*_x_/∂α of the enhancement factor *F*_x_. This leads to substantially larger band gaps^53^ than SCAN, mainly since the more negative the slope ∂*F*_x_/∂α is, the larger is Δ_xc_. The MGGAC functional^54^ was constructed using the cuspless hydrogen exchange hole in the Becke–Roussel approach.^56^ It is to be noted that the MGGAC exchange energy enhancement factor depends only on α and that the correlation component is a GGA.

**Figure 1:**
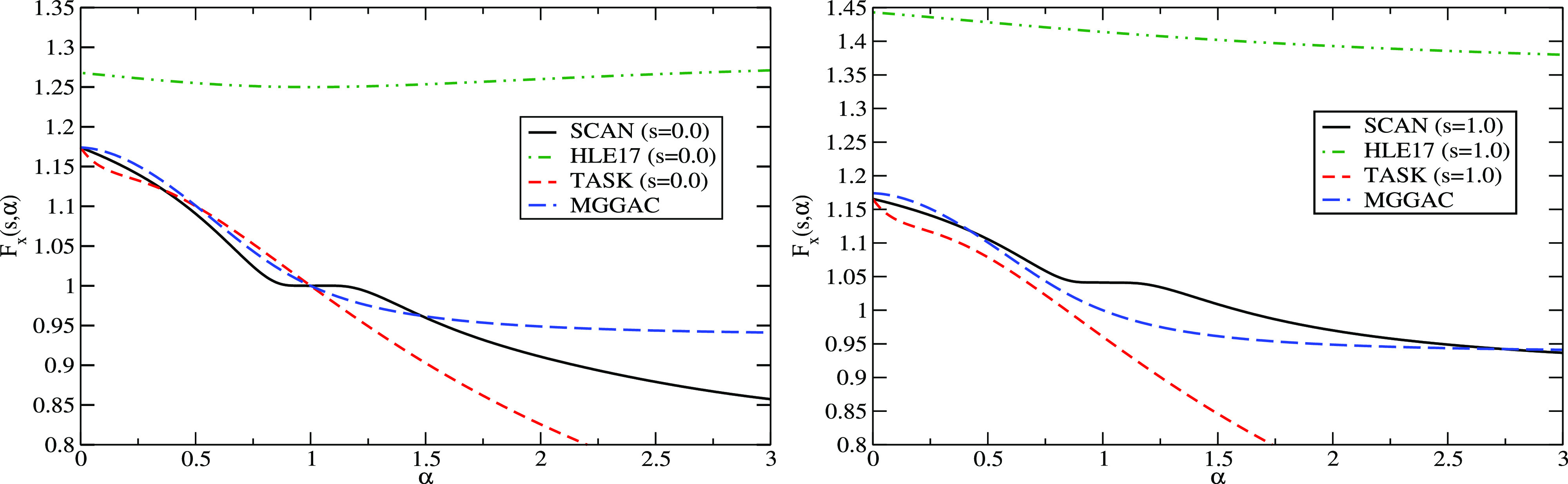
Variation of the enhancement factor *F*_x_ with α for *s* = 0 (left) and *s* = 1 (right). Four meta-GGA energy functionals SCAN, HLE17, TASK, and MGGAC are considered. Since there is no dependency on *s*, the MGGAC enhancement factor is the same in both the cases.

We plot the exchange enhancement factor of these meta-GGA functionals in Figure 1. The left panel of Figure 1 shows the variation of *F*_x_ with respect to α for *s* = 0, and the right panel shows that for *s* = 1. The HLE17 enhancement factor, which is a magnified version of TPSS, possesses a weak negative slope up to α = 1 for *s* = 0, which then becomes positive, while the slope is negative for *s* = 1. The other three functionals have roughly similar strong negative slopes up to α = 1 for *s* = 0, but then differ from each other for larger α. For TASK and MGGAC, *F*_x_ decreases monotonically, but SCAN shows a plateau for α around 1, which has been shown to be a source of numerical problems.^65^ We also note that for *s* = 1, SCAN has a weaker slope than for *s* = 0, while the opposite is observed for TASK (*F*_x_^MGGAC^ remains the same since it does not depend on *s*). Finally, we mention that the importance of the sign of the slope ∂*F*_x_/∂α in the context of magnetism has been discussed in ref (66).

There exist a plethora of other meta-GGA functionals, and among them, some others, like, for instance, revM06-L^67^ just to cite one, would probably also deserve to be considered. However, we believe that the four meta-GGA functionals that we selected are representative of the performance of meta-GGAs for band gaps.

## Computational Details

The geometry of the 2D materials was first optimized with the SCAN functional using the projector augmented wave code VASP.^68−71^ Then, the calculations of the electronic structure were done with the WIEN2k^72,73^ code, which uses the augmented-plane-wave plus local orbitals method.^74,75^ Such a common choice for the geometry allows for a study of the change in the band gap, which is only due to the XC method, i.e., without perturbation due to the different geometries. Actually, LMBJ and GLLB-SC do not allow for a geometry optimization, since these are potential-only methods and, therefore, a choice for the geometry has anyway to be made for these two methods. Also, we mention that the reported HSE06, *G*_0_*W*_0_, *GW*_0_, and *GW* collected from different papers are not calculated with the SCAN optimized geometries.

The calculations were done with parameters for the basis set, *k*-mesh, etc. which are large enough so that the results are well converged. Spin–orbit coupling (SOC) was included in the calculations involving transition-metal atoms, i.e., for the TMDs and MXenes.

## Results and Discussion

The 2D materials that we will consider are TMDs, doped (with Si or Ge) and saturated (with H, F, or Cl atoms) graphene systems, black phosphorene, and MXenes.

### Transition-Metal Dichalcogenides

The TMDs are known for their increasing applications in various fields due to their semiconducting to metallic behavior. The TMDs considered in this work, MX_2_ (M = Mo, W, Zr, or Hf and X = S, Se, or Te), possess a semiconducting band gap.^80^ The freestanding monolayers with M = Mo or W are thermodynamically stable in the 2H phase, where the central transition-metal atom is covalently bonded to the chalcogen atoms in a trigonal bipyramidal configuration. Our SCAN optimized geometries show an increase of the layer thickness as MS_2_ < MSe_2_ < MTe_2_, along with increasing bond length between the transition-metal and chalcogen atoms. These 2D materials have a direct gap (the bulk counterparts have an indirect band gap) that makes them more relevant for optoelectronic and photovoltaic cell applications.^81^ The direct band gap is attributed to the absence of in-plane inversion symmetry.^80^ The monolayers with M = Zr or Hf are stable in the octahedral 1T phase and possess an indirect fundamental band gap.

As shown in Table 1, the band gap decreases with the mass of the X atom with any method. Regarding the two GGA functionals PBE and HLE16, PBE leads to a larger (by 0.1–0.15 eV) band gap when the M atom belongs to group 6 of the periodic table (Mo or W), and therefore to a better (but still very bad) agreement with the HSE and *G*_0_*W*_0_ results. This is rather surprising since the HLE16 band gap is larger than the PBE one for basically all bulk solids (see, e.g., ref (29)). However, when M belongs to group 4 (Zr or Hf), the band gaps are clearly larger with HLE16 than with PBE, as normally expected.

**Table 1:** Band Gaps (in eV) of Monolayers of TMDs^f^

TMD	PBE	HLE16	LMBJ	GLLB-SC(Δ_x_)	SCAN	HLE17	TASK	MGGAC	HSE	*G*_0_*W*_0_	expt.	expt.
MoS_2_ (2H)	1.62	1.47	1.60	**2.81**(1.19)	1.72	1.48	1.79	1.65	2.05^a^, 2.09^b^	2.82^a^, 2.54^b^	2.50^c^	1.83^d^
MoSe_2_ (2H)	1.37	1.24	1.36	**2.42**(1.05)	1.47	1.24	1.54	1.41	1.75^a^, 1.80^b^	2.41^a^, 2.12^b^	2.31^c^	1.66^d^
MoTe_2_ (2H)	1.01	0.91	1.03	**1.87**(0.85)	1.11	0.92	1.14	1.07	1.30^a^, 1.37^b^	1.77^a^, 1.56^b^		1.10^d^
WS_2_ (2H)	1.58	1.43	1.68	**2.64**(1.06)	1.66	1.48	1.79	1.71	1.87^a^, 2.06^b^	2.88^a^, 2.53^b^	2.72^c^	1.95^d^
WSe_2_ (2H)	1.31	1.16	1.38	1.32(0.04)	1.37	1.20	**1.49**	1.42	1.68^a^, 1.73^b^	2.34^a^, 2.10^b^		1.64^d^
WTe_2_ (2H)	0.82	0.71	0.89	0.82(0.04)	0.88	0.75	**0.97**	0.93	1.14^b^	1.79^a^,1.38^b^		
ZrS_2_ (1T)	1.20	1.51	1.80	**2.43**(0.70)	1.56	1.55	2.00	1.86	2.17^b^	2.89^b^		
ZrSe_2_ (1T)	0.42	0.69	0.92	**1.21**(0.35)	0.76	0.74	**1.21**	1.08	1.20^b^	1.69^b^		
HfS_2_ (1T)	1.22	1.73	2.10	**2.37**(0.69)	1.56	1.69	2.08	1.91	2.15^b^	2.94^b^		
HfSe_2_ (1T)	0.45	0.88	1.19	1.18(0.34)	0.77	0.85	**1.31**	1.14	1.22^b^	1.79^b^		1.1^e^

a Ref (76).

b Ref (42).

c Ref (77).

d Refs (48, 78).

e Ref (79).

f The values for HSE and *G*_0_*W*_0_ are from the literature. Direct optical band gaps are also shown (the results from the first column are free from the excitonic effect). The values of the semilocal methods which agree the best with the *G*_0_*W*_0_ values are in bold.

When M belongs to group 6, in most cases, the band gaps obtained with the meta-GGA energy functionals can be ordered as HLE17< SCAN < MGGAC < TASK, and only TASK leads to values clearly larger than PBE. However, they are still much smaller than the HSE and *G*_0_*W*_0_ values. Also surprising, the LMBJ potential leads to band gaps that are quasi-identical to PBE (differences in the range of 0.01–0.05 eV). Therefore, LMBJ is inferior to the meta-GGA functionals except HLE17, the latter giving band gaps very similar to HLE16. When M belongs to group 4, the SCAN and HLE17 band gaps are the same for M = Zr, but slightly larger with HLE17 for M = Hf. The TASK values are still the largest among the meta-GGA energy functionals. Another difference with respect to the case of group 6 M atoms is that the LMBJ band gaps are much larger by at least 0.5 eV than the PBE values.

For many of the systems, the GLLB-SC band gaps are the largest and therefore much closer to the *G*_0_*W*_0_ values, in agreement with ref (42). Thanks to the explicit inclusion of the exchange derivative discontinuity Δ_x_ (mentioned within parentheses in Table 1), the GLLB-SC band gaps are greatly enlarged except for WSe_2_ and WTe_2_, which have a small Δ_*x*_. However, for some of the systems (WSe_2_, WTe_2_, and HfSe_2_), methods like TASK or LMBJ lead to larger band gaps.

**Figure 2:**
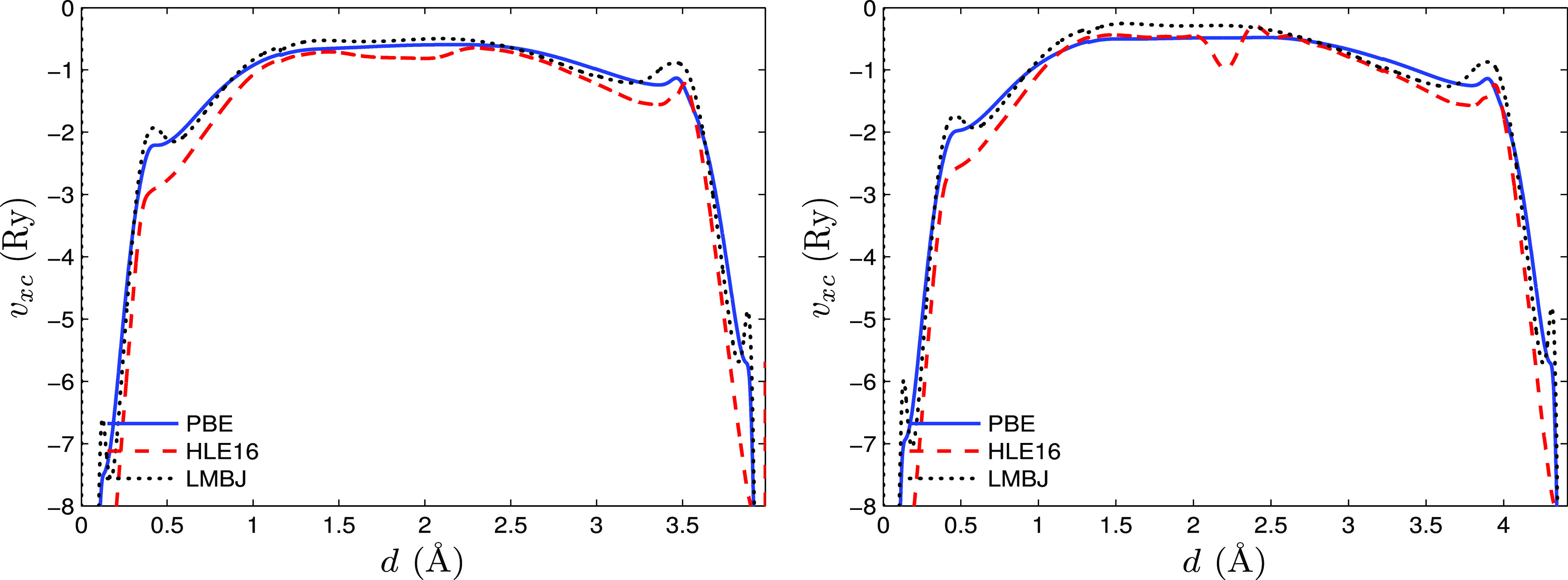
PBE, HLE16, and LMBJ potentials in MoS_2_ (left) and ZrS_2_ (right) plotted along the path from an M atom (at *d* = 0) to the second-nearest-neighbor S atom.

The PBE, HLE16, and LMBJ potentials along the path from an M atom to a second-nearest-neighbor S atom in MoS_2_ and ZrS_2_ are compared in Figure 2. As mentioned above, the PBE and LMBJ band gaps are very close in MoS_2_, which is consistent with the fact that the PBE and LMBJ potentials are more similar in the case of MoS_2_ than ZrS_2_. In the middle region between the M and S atoms (slightly toward the M atom on the left), the LMBJ potential is less negative than the PBE potential in a more pronounced way in the case of ZrS_2_.

Concerning the HSE band gaps, we note that they are significantly smaller than the *G*_0_*W*_0_ values, which is a general observation for all systems studied in this work. The same trend has been obtained in the recent work by Rauch et al.^47^ on a set of 298 2D materials.

A comparison with experimental values that were obtained from optical measurements needs to be done by taking into account the excitonic effect and lattice polarization, which reduce the band gap.^38,82^ Although the excitonic effect, which is an effect beyond DFT and *GW* (without vertex correction), is usually small in bulk solids (of the order of tens of meV), it can reach several electronvolts in 2D systems or molecules (see, e.g., ref (83)). It is in the range 0.4–1 eV for the TMDs according to the Bethe–Salpeter equation (BSE) calculations from refs (42, 76). The band gaps obtained with the meta-GGA functionals TASK, SCAN, and MGGAC agree (incidentally) quite well with the optical gap, as shown in Table 1. The values from the first “expt.” column are estimations of the band gap without the excitonic effect (see ref (77) and references therein) and agree very well with the *G*_0_*W*_0_ results.

### Si- and Ge-Doped Graphene

To apply graphene-based systems in optoelectronic devices, the band structure needs to be engineered to get semiconducting band gaps. The isovalent atom doping in graphene opens the band gap at the high-symmetry *K* point^6,26,85^ due to symmetry breaking in the graphene lattice. In this regard, the doping of graphene with Si and Ge opens the band gap, and the values depend on the doping percentage. As in refs (6, 26, 84), we consider 50, 25, 12.5, and 8.33% Si/Ge-doping of graphene. It has to be noted that in ref (6), we showed that except for the 50% doping case, the Ge-doped graphene structures are dynamically unstable. Nevertheless, we can still employ these systems to assess DFT methods, since results from more advanced methods (HSE and *GW*) are available. In Tables 2 and 3, we show the DFT band gaps for the Si- and Ge-doped graphene systems, respectively.

**Table 2:** Band Gaps (in eV) of Si-Doped Graphene at Different Doping Percentages^a^

doping (%)	PBE	HLE16	LMBJ	GLLB-SC(Δ_x_)	SCAN	HLE17	TASK	MGGAC	HSE	*GW*_0_	*GW*
50	2.57	2.89	3.20	4.57(1.41)	2.87	2.94	**3.48**	3.40	3.42	3.88	4.10
25	1.31	1.47	1.78	**2.42**(0.72)	1.55	1.51	1.73	1.80	1.83	2.27	2.51
12.5	0.77	0.88	1.10	**1.44**(0.42)	0.93	0.91	1.02	1.09	1.13	1.46	1.69
8.33	0.53	0.61	0.74	**0.97**(0.28)	0.64	0.63	0.70	0.75	0.79	0.92	1.11

a The band gaps for HSE are from ref (26), and the values for *GW*_0_ and *GW* are from ref (84). The values of the semilocal methods which agree the best with *GW*_0_/*GW* values are in bold.

**Table 3:** Band Gaps (in eV) of Ge-Doped Graphene at Different Doping Percentage^a^

doping (%)	PBE	HLE16	LMBJ	GLLB-SC(Δ_x_)	SCAN	HLE17	TASK	MGGAC	HSE
50	2.11	2.47	2.78	3.82(1.13)	2.22	2.41	2.66	**2.80**	2.79
25	0.95	1.34	**1.51**	1.79(0.53)	1.11	1.31	1.04	1.37	1.58
12.5	0.67	0.82	**0.98**	1.27(0.37)	0.77	0.82	0.86	**0.96**	1.00
8.33	0.48	0.58	0.69	0.88(0.25)	0.53	0.58	0.61	**0.68**	0.72

a The values of the semilocal methods which agree the best with HSE values are in bold.

#### Silicon-Doped Graphene

It is observed that the band gap increases with the doping percentage, and all methods follow this trend. The GGA functionals PBE and HLE16 underestimate the gaps drastically compared to *GW*_0_ or *GW*. Here, HLE16 leads to larger band gaps than PBE, while it was the reverse for some of the TMDs. Among meta-GGA energy functionals, both SCAN and HLE17 underestimate the band gap. In contrast, the MGGAC and TASK functionals predict band gaps quite similar to the hybrid HSE method, and the underestimation with respect to the *GW* methods is not as pronounced as for the TMDs. Yet another important difference with respect to the TMD monolayers is the very close similarity between the LMBJ and HSE band gaps. Concerning the GLLB-SC potential, we observe that the band gap of 4.57 eV for 50% doping is clearly larger than the *GW* values, but in the other three cases, GLLB-SC band gaps are in very good agreement with *GW*_0_ and *GW*. Thus, in this case, the GLLB-SC potential is more reliable than the LMBJ, while the MGGAC functional and the LMBJ potential are as accurate as HSE.

#### Germanium-Doped Graphene

The trends observed for Ge-doped graphene are basically the same as for Si-doped graphene. For instance, PBE (GLLB-SC) leads to the smallest (largest) band gaps, while LMBJ, MGGAC, and HSE lead to similar values. Though we do not have any *GW* values for Ge-doped graphene, it is however reasonable to suppose that the GLLB-SC potential should be the closest to *GW* as in the case of Si-doped graphene.

### Hydrogen-, Fluorine-, and Chlorine-Saturated Graphene

Besides substitution of carbon with isovalent atoms, the semimetallic band gap of graphene can also be modified to a semiconducting band gap by saturating the extra electron present at each carbon site.^6^ This is possible by terminating each carbon atom with an atom missing an electron to complete the outer shell, e.g., hydrogen or halogens such as fluorine or chlorine. The saturated graphene systems have two conformations, chair-like and boat-like. In both conformations, the planar geometry of graphene changes to a buckled structure by forming a covalent bond at each carbon atom and the hybridization changes to sp^3^. The band gaps of these systems are tabulated in Table 4.

**Table 4:** Band Gaps (in eV) of H-, F-, and Cl-Saturated Graphene in Chair and Boat Conformations^a^

system	PBE	HLE16	LMBJ	GLLB-SC(Δ_x_)	SCAN	HLE17	TASK	MGGAC	HSE	*G*_0_*W*_0_	*GW*_0_	*GW*
Chair Conformation												
H-graphene	3.41	4.82	5.06	6.91(1.80)	4.02	**4.90**	4.53	4.57	4.37	5.64	5.89	6.28, 5.4
F-graphene	3.18	4.05	**5.88**	5.08(1.22)	3.75	4.49	4.00	4.22	4.91	6.98	7.48	8.12
Cl-graphene	1.56	1.85	2.53	2.43(0.61)	1.91	1.93	1.91	2.20	**2.85**	4.07	4.46	4.89
Boat Conformation												
H-graphene	3.29	4.72	6.12	6.51(1.48)	3.91	**4.80**	4.34	4.45	4.28			5.10
F-graphene	3.17	4.22	**5.87**	5.18(1.26)	3.70	4.60	4.02	4.14	4.89		5.68	

a The HSE, *G*_0_*W*_0_, *GW*_0_, and *GW* results from refs (86−88) are also listed. The values of the semilocal methods which agree the best with *G*_0_*W*_0_/*GW*_0_/*GW* values are in bold.

#### Graphene Saturated with Hydrogen

The chair conformation of hydrogenated graphane, known as graphene, is energetically more stable than the boat conformation,^89^ and the band gap of chair-like geometry is larger than the boat-like geometry. The HLE16 band gaps are much larger (by 1.4 eV) than that of PBE in both conformations. HLE16 leads to larger band gaps than all of the meta-GGA energy functionals except HLE17. This is in contrast to the results from the previous sections, where HLE16 and HLE17 were providing rather small band gaps compared to the other GGAs and MGGAs. As for Si/Ge-doped graphene, the GLLB-SC band gaps are by far the largest among the semilocal methods; however, here, a clear overestimation with respect to the *GW* results is obtained. The LMBJ potential is along with GLLB-SC the most accurate for the chair conformation, but clearly overestimates for the boat conformation. Keeping in mind that the full *GW* method overestimates, *G*_0_*W*_0_ underestimates, and *GW*_0_ should be closer to experimental values,^37^ HLE17 seems to be the best semilocal method for H-saturated graphene.

#### Graphene Saturated with Fluorine

Both conformations of fluorinated graphene are dynamically stable and possess insulating band gaps.^87^ From Table 4, it can be observed that the HSE, *GW* (all three variants), and LMBJ band gaps of fluorinated graphene are larger than those of graphane. However, all other methods predict smaller band gaps than graphane. This is an interesting difference in the trends between HSE/*GW*/LMBJ and the other methods. Furthermore, from H-graphene to F-graphene, the increase or decrease of the band gap can be quite substantial (e.g., +0.8 eV with LMBJ and at least −1.5 eV with *GW*). Another observation is that the band gaps of the chair and boat conformations are very close to each other, except with *GW*_0_. Among the semilocal GGA and meta-GGA energy functionals, the HLE17 band gaps are the closest, but still too small, to the *GW*_0_ values. However, the LMBJ potential has the largest band gaps among all of the DFT (including GLLB-SC and HSE) methods and is the most accurate. Except PBE and SCAN, all other methods are able to satisfy the lower limit set by the photoemission measurements, i.e., >3.8 eV.^90^

#### Graphene Saturated with Chlorine

The boat conformation of chlorinated graphene is unstable;^91^ thus, only the band gap of the chair-like geometry is considered. As observed from Table 4, none of the energy functionals or potentials are close to the quasi-particle gaps. All semilocal methods underestimate the various *GW* values by at least 1.5 eV. The HSE band gap is slightly larger, 2.85 eV, but still too small compared to the *GW* values that are above 4 eV.

**Figure 3:**
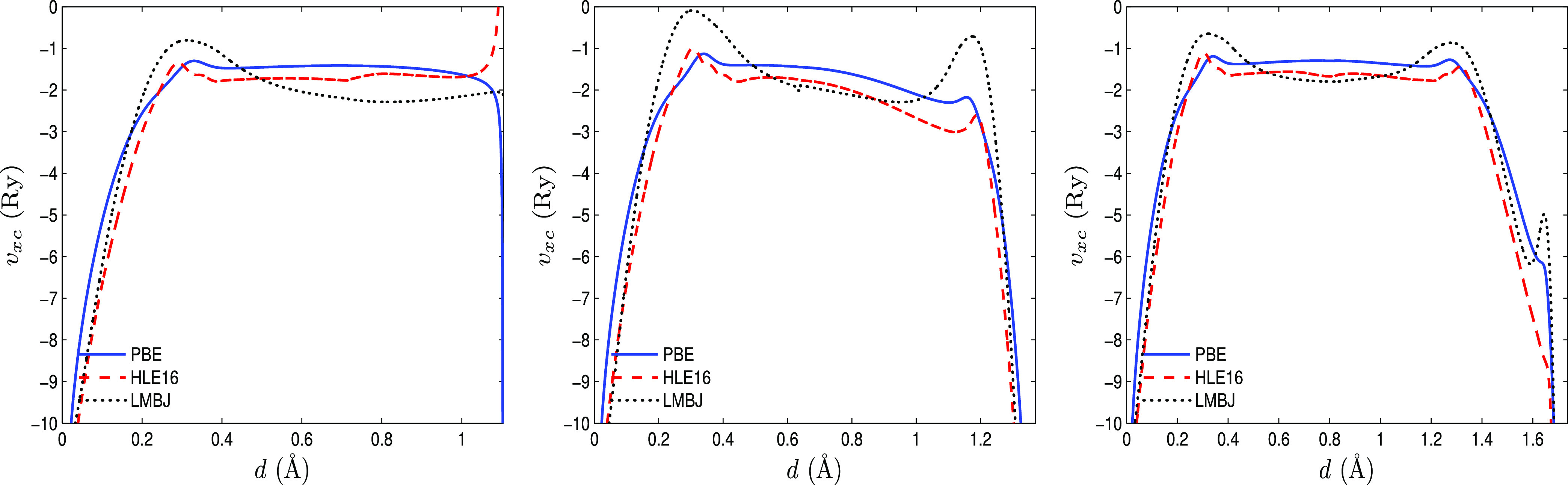
PBE, HLE16, and LMBJ potentials in the chair conformation of H-graphene (left), F-graphene (middle), and Cl-graphene (right) plotted along a C–X (X = H, F, Cl) bond.

We can see that LMBJ leads to a much larger band gap than HLE16. The same is observed for F-graphene but to a much lesser extent for H-graphene. To understand this difference between H-graphene and the two other systems, Figure 3 compares the PBE, HLE16, and LMBJ potentials along a C–X (X = H, F, Cl) bond. A major difference can be seen; in H-graphene, the HLE16 potential goes to positive values close to the H atom, while the LMBJ potential stays rather constant and PBE goes to negative values. For the two other systems, the LMBJ potential shows the least negative values on the X atom. Thus, these different behaviors should affect the band gap and explain the observed trends.

### Phosphorene Layers

Black phosphorus (BP) is one of the thermodynamically stable allotropes of phosphorus and possesses a narrow band gap of 0.3 eV.^93^ The electronic structure of one-layer-thick black phosphorus, known as phosphorene, has been studied both experimentally and theoretically.^8,92−94^ A single-layer unit cell of phosphorene contains four phosphorus atoms in a puckered honeycomb lattice, and each sp^3^-hybridized phosphorus atom is covalently bonded to adjoining atoms. The change in the electronic structure with respect to the number of layers has been discussed in refs (92, 93). For the present work, we have calculated the band gap of phosphorene for a number of layers varying from one to four with the semilocal methods and HSE. *GW*_0_ results as well as values from optical absorption experiments are listed in Table 5. As in the case of the graphene-based systems discussed in the previous sections, HLE16 band gaps are larger than the PBE values and quite similar to its meta-GGA counterpart HLE17. MGGAC results are relatively similar to HLE16 and HLE17; however, SCAN and TASK band gaps are smaller and intermediate between PBE and HLE16 values. Compared to the *GW*_0_ values, all DFT band gaps are smaller except for the four-layer case for which HLE16, HLE17, and HSE gaps are slightly larger. Comparing now to the experimental optical gap, the GLLB-SC values are the closest, while other methods like MGGAC, LMBJ, and HSE also lead to similar values depending on the number of layers. Note that in the case of the TMDs, the GLLB-SC band gaps were much larger (by up to 1 eV) than the optical gaps.

**Table 5:** Band Gaps (in eV) of Mono- and Multilayer Phosphorene^d^

no. layers	PBE	HLE16	LMBJ	GLLB-SC(Δ_x_)	SCAN	HLE17	TASK	MGGAC	HSE	*GW*_0_	expt.^c^
1	0.91	1.38	1.50	**1.75**(0.55)	1.17	1.45	1.26	1.52	1.60, 1.51^a^	1.94^b^, 2.03^a^	1.73
2	0.57	1.12	1.09	**1.18**(0.37)	0.81	1.15	0.90	1.13	1.20	1.65^b^	1.15
3	0.37	0.96	0.86	0.85(0.26)	0.59	**0.98**	0.69	0.91	0.97	1.37^b^	0.83
4	0.28	0.90	0.75	0.70(0.22)	0.50	0.90	0.60	**0.81**	0.87	0.82^b^	

a Ref (42).

b Ref (92).

c Ref (93).

d Direct optical band gaps are also shown. The values of the semilocal methods which agree the best with *GW*_0_ values are in bold.

### Monolayer of MXenes

Besides graphene-based materials and transition-metal dichalcogenides, transition-metal carbides or nitrides are also emerging materials having a wide range of applications.^96^ These 2D systems, known as MXenes, are synthesized by etching from the corresponding precursor MAX phases,^97−100^ where M = (Sc, Ti, V, Cr, Zr, Nb, Mo, Hf, or Ta), A = (Cd, Al, Si, P, S, Ga, Ge, As, In, Sn, Tl, Pb, or S), and X = (C and/or N). In the etching process, the A atom is washed out and the outer layers are saturated with oxygen, fluorine, and/or hydroxyl group. The general formula for MXenes is written as M_*n*+1_X*_n_T*_z_, where *T*_z_ is the termination mentioned above.^101^ Depending upon the type of transition metal and surface termination, an MXene can behave as a metal or as a semiconductor.^100,102^ For the present study, we have considered three semiconducting MXenes. They correspond to M = Ti, Zr, or Hf and X = C with an oxygen-saturated surface.^95,100^ In addition to our results, shown in Table 6, available HSE and quasi-particle (*GW*) band gaps are also reported. From the *G*_0_*W*_0_ and HSE methods, it is clear that the band gap decreases with the increase of transition-metal atom mass. However, the PBE and SCAN functionals and the GLLB-SC potential do not follow this trend. Instead, for these three methods, the band gap of Zr_2_CO_2_ is, by a small amount, larger than that of Hf_2_CO_2_. With MGGAC, the band gap is the same for these two systems. The band gaps obtained with GLLB-SC are the closest to the *G*_0_*W*_0_ values, and actually larger than the HSE band gaps for the three systems. The LMBJ band gaps are overall also slightly larger than the HSE values. Among the meta-GGA energy functionals, TASK leads to more accurate band gaps than the others, and the MGGAC comes as the second best.

**Table 6:** Band Gaps of MXenes^a^

MXenes	PBE	HLE16	LMBJ	GLLB-SC(Δ_x_)	SCAN	HLE17	TASK	MGGAC	HSE	*G*_0_*W*_0_
Ti_2_CO_2_	0.33	0.42	0.92	**0.96** (0.23)	0.52	0.51	0.71	0.66	0.90	1.15
Zr_2_CO_2_	0.96	1.13	1.54	**1.95** (0.52)	1.20	1.17	1.51	1.39	1.45	2.13
Hf_2_CO_2_	0.94	1.24	1.84	**1.93** (0.51)	1.16	1.24	1.58	1.39	1.59	2.45

a The HSE and *GW* band gaps are from ref (95). The values of the semilocal methods which agree the best with *G*_0_*W*_0_ values are in bold.

## Summary and Conclusions

In this work, various DFT semilocal methods have been applied to 2D materials. The goal was to assess the accuracy of the considered DFT methods for band gap calculations, more particularly LMBJ, which has been developed very recently. *GW* quasi-particle band gaps from the literature were used as a reference. The main observations are the following. The meta-GGA functionals are usually more accurate than the standard PBE GGA, but with some exceptions like with HLE17 for some of the TMDs. Among the meta-GGA functionals, HLE17 band gaps are sometimes the least accurate, sometimes the most accurate, making HLE17 quite unpredictable. It is observed that the SCAN functional does not have a single case among considered structures, where it agrees best with the reference value. Therefore, TASK and MGGAC should be considered as more reliable meta-GGA functionals for band gap calculations for 2D materials; however, neither of them shows a systematic good performance. Regarding methods that consist of only a potential (no energy functional), GLLB-SC band gaps are larger than the LMBJ values in the majority of cases, thus in better agreement with the quasi-particle *GW* band gaps. The LMBJ potential shows good performance more or less only for the systems based on graphene; thus, in 2D materials, LMBJ does not seem to retain the dominance of the MBJ potential for bulk materials. By analyzing all of the studied cases, it is clear that the band gap prediction can be successful within the semilocal approximations and GLLB-SC seems to be a pretty good choice for such calculation, however by far not systematically. It would be interesting to test other functionals for 2D materials, and if performance is not better than those tested here, then more attempts should be made to improve the exchange potentials. Concerning HSE, it is very accurate for bulk materials with small band gaps (semiconductors), but not so for 2D systems since the band gaps are very often significantly smaller gaps than the *G*_0_*W*_0_ predictions.
